# Correspondence

**Published:** 1856-06

**Authors:** 


### Correspondence.

Saint Louis, June, 1856.

Dear Sir:—An adjourned meeting of the Western Dental Society, is to be held in the city of Chicago, commencing on Wednesday, July 30th, at 10 o'clock, A. M.

The object of the meeting is to continue the work which had so favorable a beginning at the meeting held in this city in April last, by extending the membership and operations of the Society over that portion of the West which has not been hitherto embraced in any similar organization.

The invitation is intended to be general, and it is hoped that no one will feel himself neglected in case he does not receive a copy of this Circular; but that each and all will interest themselves in the movement, and give it their hearty co-operation and support.

The place of meeting can be ascertained by inquiry at any of the principal Hotels in Chicago.

Very Respectfully, Yours,
C. W. Spalding,
Corresponding Secretary.

We give place to the notice of the Western Dental Society, which we hope will be well attended.—Ed.

---

Dr. James Taylor—Dear Sir—As it is a desirable object to the profession, to obtain information relative to the best mode of getting up atmospheric plates, I beg leave to lay before you a manner of procedure practised by me the past year, with the utmost satisfaction; if you think it worthy a place in your Journal, it may benefit a great many brethren.

In taking the impression of the mouth I forego the use of wax, and in its place use gypsum, it is more cleanly, and less objectionable to the patient. I find the Ohio plaster sets more readily than any other, it not taking more than three to five minutes to become hard; when you have an impression in this way, there are no imperfections in it; previous to taking a plaster model for moulding, you should slightly cover with a thin coat of varnish, or saturate well with sweet oil, so there may be no difficulty in drawing it; having obtained your model, take a hair pencil, and with a thin paste of whiting, build up all the small prominences which are upon the arch, also around the alveolar ridge. This is to guard against the battering of these prominences upon the zinc cast in stamping the plate, which generally occurs, and gives rise to rocking, so much complained of; should there be a slight vacuum between these prominences, it will do no harm, it will rather assist in giving a suction; when your model is sufficiently dry varnish the whole, then take a piece of sheet lead, and form it in shape as you would like your air chamber, press it closely upon your model with your fingers, being soft and pliable, you will not injure it, fasten with wax cement. Mould in sand and cast two dies, one of them is to be kept in reserve for a finishing touch, the other you take a counter model of lead, and proceed to bring up your plate as accurately as possible, in doing so, you should chase around the chamber with a small brass tool, and anneal frequently; when this is done you should place your plate upon your reserved die, slightly invest it with the paste of whiting; when dry, cast lead over, and you have another set of dies more perfect. After it has become cool, separate, cleanse the dies and plate and restamp, when you will have a perfect suction plate. The next thing is to guard against shrinkage in soldering, which may be done in the following manner: well anneal your plate, adjust the teeth, take a piece of fine Russia sheet iron cut in shape of your case, three-quarters of an inch wide, clip both edges in points with a pair of shears, turn the inner points so as to drop down into the alveolar ridge, turn the outer points so as to lay over and embrace the teeth, then anneal your iron piece, invest a moderate quantity of plaster between, around and over the whole, dry gradually, heat up slowly in a charcoal fire, and solder quick, and give plenty of time to cool, and success is sure to follow. I always back up my teeth with continuous plate, use small pieces of solder, and little borax, but it should be clean and tolerable thick; too much borax is detrimental, it causes shrinking.

Respectfully Yours,

J. Owen, Dentist,

June 18th, 1856. No. 36 West Sixth st.

 Cincinnati.

